# Impact of quality of life related to foot problems: a case–control study

**DOI:** 10.1038/s41598-021-93902-5

**Published:** 2021-07-15

**Authors:** Daniel López-López, Mónica Pérez-Ríos, Alberto Ruano-Ravina, Marta Elena Losa-Iglesias, Ricardo Becerro-de-Bengoa-Vallejo, Carlos Romero-Morales, Cesar Calvo-Lobo, Emmanuel Navarro-Flores

**Affiliations:** 1 Departamento de Medicina Preventiva e Saúde Pública, Facultad de Medicina e Odontoloxía, Santiago de Compostela, Spain; 2 Grupo de Investigación Saúde e Podoloxía, Departamento de Ciencias da Saúde, Facultade de Enfermaría e Podoloxía, Universidade da Coruña, Campus Universitario de Esteiro s/n, 15403 Ferrol, Spain; 3 CIBER de Epidemiología y Salud Pública, CIBERESP, Madrid, Spain; 4 Faculty of Health Sciences, Universidad Rey Juan Carlos, Alcorcón, Spain; 5 School of Nursing, Physiotherapy and Podiatry, Universidad Complutense de Madrid, Madrid, Spain; 6 Faculty of Sport Sciences, Universidad Europea de Madrid, Villaviciosa de Odón, Madrid Spain; 7 Department of Nursing, Faculty of Nursing and Podiatry, Frailty Research Organized Group, Universidad de Valencia, Valencia, Spain

**Keywords:** Health care, Medical research

## Abstract

Foot problems are highly prevalent conditions, being a frequent reason for medical and podiatric consultation. The aim of this study was to compare the differences of quality of life (QoL) related to foot health in people with and without the presence of foot problems. A case–control study was carried out in an outpatient centre, where a clinician recorded data related to sociodemographic and clinical characteristics. In addition, self-reported data on foot health-related quality of life were recorded using the Spanish version of the Foot Health Status Questionnaire. The sample consisted of 498 participants (249 cases and 249 controls), with a median age of 30 years and an interquartile range of 23 years. The differences between the groups were statistically significant for gender, age, footwear, general health, foot health, and physical activity. Cases showed lower scores for the domain of footwear, physical activity and vitality compared to controls. Foot pathologies have a negative impact on quality of life related to foot health, and the domains of footwear, general health and physical activity seem to be the factors that are associated with the presence of alterations and foot deformities.

## Introduction

The feet are an essential foundation of people's health, and due to their complex anatomical characteristics, they play a key role in posture and ambulation, since they are responsible for the autonomy, independence and well-being of the individual. Currently, there is an increase in the prevalence of foot pathologies, ranging between 61 and 79%, which is why they constitute an important public health problem^1,2^. In addition, there are other factors, such as the difficulty in managing foot problems, in part due to their multifactorial aetiology, the discomfort they can cause, and the high demand regarding these complaints by patients^2,3^, that could result in their chronicity.

Moreover, the non-existence of two identical cases among people who suffer from them should be underlined, as they are determined by a specific diagnosis, and the characteristics of the structures involved, whether they are: ligamentous, muscular, bone, vascular and/or nervous^4,5^, producing an increase in health spending and a worsening of established cases. Thus, foot problems can reduce quality of life, lead to loss of balance, make it difficult to put on shoes, and increase the risk of falling^6–8^. All of this can affect activities of daily living, including the desire to go outside.

Despite the importance of foot pathologies, both due to their prevalence and their impact on activities of daily living, there are few studies in Spain that have measured the influence of quality of life in these patients compared to healthy subjects and, specifically, which facets of quality of life could be most affected by these pathologies^9–11^.

The objective of this study was to compare quality of life related to foot health in people with and without the presence of foot problems.

## Material and methods

### Design and sample

This is a case–control study carried out in a private podiatry centre providing foot health care services, in the city of A Coruña (Galicia, Spain), between January 2016 and December 2017. The selection of study participants was carried out through non-random sampling and the recommendations for the communication of observational studies, known as Strengthening the Reporting of Observational Studies in Epidemiology (STROBE)^12^, were followed.

The inclusion criteria for the group of cases were established as interest in participating and completing the study phases. The exclusion criteria were the following: immunosuppressed people, people with the presence of systemic conditions, a history of surgery and / or orthopaedic treatments on the feet, lack of partial or total autonomy in daily activities, as well as those who did not want to sign the consent form or did not understand the instructions to participate in the research. In the group of controls, the inclusion criteria were the following: people who attended for a health check on their feet, who did not present any problems with them and who completed all phases of the investigation. Regarding the exclusion criteria of the control group, they were related to: presenting foot pathologies, drug use, presenting any systemic disease or inability to carry out the research. All participants had a median age of 30 years, an interquartile range of 23 years, with no upper age limit, and there were no criteria established by gender. All subjects signed the informed consent to be included in the study.

### Calculation of the sample size

The sample size required for this case–control study, with specific levels of confidence, power, and groups of equal size was calculated through the Epidat version 4.2 programme (Consellería de Sanidade, Xunta de Galicia, Spain; Organización Panamericana de la salud (OPS-OMS); Universidad CES, Colombia).

A total sample size of 498 subjects (249 per group) was determined assuming a confidence level of 95%, a power of 0.80, an odds ratio to detect of 2.0 and an expected proportion of exposed of 88.268%, and in the controls of 79%. The actual sample (total of 498 participants) consisted of 249 cases (15 men and 234 women) and 249 controls (45 men and 204 women).

This research was approved by the Research Ethics Committee of the University of Coruña, with file number CE 010/2015. All participants were informed about the procedures used in this study to give and sign their informed consent. Additionally, the guidelines associated with the ethical standards for investigation and experimentation in people as reported in the Declaration of Helsinki, in their last modification and others internationals institutionals organisations bodies were preserved.

### Procedure

In the first place, an experienced clinician with more than ten years’ providing attention to the treatment of foot pathologies, recorded the general health status of each patient, the anthropometric variables (age, sex, body mass index), the medical and surgical history, the presence of systemic diseases and current medication.

Second, a physical examination of the general state of the health of the feet was carried out by means of structural assessment using palpation, analysis of joint mobility and tests of muscle strength in the foot. In addition, the clinical history of each patient was accessed to verify any other foot pathology and / or chronic diseases, as well as complementary tests (ultrasound and X-rays).

Third, for the evaluation of the impact of quality of life related to foot health, each patient anonymously self-administered the Foot Health Status Questionnaire, in its Spanish version^13^. This tool contains three sections. The first section presents a high degree of validity related to the content, the evaluation criteria and the construct of the four specific domains to analyse foot health associated with: foot function, foot pain, footwear and condition of foot health, with a Cronbach's alpha of 0.89–0.95, and high retest reliability with an intraclass correlation coefficient of 0.74–0.92^14^. The second section was validated and adapted from the Medical Outcomes Study 36-Item Short-Form Health Survey and presents four domains for the assessment of general health, physical function, social function, and finally vitality^15,16^. The third section contains the record of the sociodemographic characteristics corresponding to the clinical history of each participant^16^. Once the third phase was completed by the patient, the clinician recorded the information from each questionnaire using the FHSQ software (version 1.03). This programme provides the final score for each dimension in a range from 0 to 100, with zero identifying the worst state of health and 100 an optimal state of health.

### Statistical analysis

The anthropometric variables (age, sex, body mass index) and the independent variables were presented as mean and standard deviation (SD) and with the ranges of maximum and minimum values. Regarding the categorical variables, they were presented with absolute values ​​and with percentages. Fisher's exact test was used to test the differences in the frequencies of the levels of categorical variables (sex) between the groups with and without foot pathologies, while the independent sample t-test was used to test the differences between the two groups in the form of continuous variables (age, height, weight, BMI, foot pain, foot function, footwear, foot health, general health, physical activity, social function, vitality). Differences were considered significant when the p value < 0.05. A multivariate logistic regression was performed to predict the case or control status based on the scores of the items on the scale considered. For this, and to have greater power, each item was divided into two categories, using the median as the cut-off point and considering the category with the best result as the reference category. Results were adjusted for sex, age, and BMI and are provided as ORs with 95% confidence intervals. Data were processed with the statistical package IBM SPSS Statistics version 25.

## Results

A total of 498 participants completed the investigation, 249 cases and the same number of controls. Regarding the age distribution of the sample, it ranged from 15 to 69 years, with a median age of 30 years, and an interquartile range of 23. The sample included 88% of women. The composition of the sample can be seen in Table 1.

**Table 1:** Comparison of demographic characteristics of the total sample, patients with foot problems and controls.

	Total group Mean ± SD Range (n = 498)	Cases Mean ± SD Range (n = 249)	Controls Mean ± SD Range (n = 249)
Weight (kg)	67.02 ± 14.18 (40–121)	65.04 ± 13.97 (40–121)	69.01 ± 14.15 (43–120)
Height (cm)	1.65 ± 0.08 (1.50–1.98)	1.64 ± 0.07 (1.50–1.93)	1.66 ± 0.08 (1.50–1.98)
BMI (kg/m^2^)	24.64 ± 4.78 (16.80–43.51)	24.17 ± 4.84 (16.80–43.18)	25.11 ± 4.68 (17.30–43.51)
**Sex (%)**			
Male	60 (12%)	15 (6%)	45 (18.1%)
Female	438 (88%)	234 (94%)	204 (81.9%)

*BMI* body mass index, *SD* standard deviation.

Regarding the results on quality of life related to foot health among the group of cases and controls, they are shown in Table 2. These scores were higher for the control group, in the first section for the footwear domain and lower for foot pain, foot function, and overall foot health. In the second section, they obtained higher scores in the domains of physical activity and vitality and lower scores in the domains of general health and social capacity.

**Table 2:** Comparison of FHSQ scores of the total sample, patients with foot problems and controls.

	Total Group (n = 498)	Cases (n = 249)	Controls (n = 249)	*p* value
Foot pain	76.81 ± 19.75 (0–100)	78.36 ± 19.07 (0–100)	75.25 ± 20.32 (0–100)	.082†
Foot function	82.68 ± 19.52 (0–100)	82.99 ± 18.24 (6.25–100)	82.36 ± 20.76 (0–100)	.763†
Footwear	47.20 ± 30.84 (0–100)	42.03 ± 31.99 (0–100)	52.37 ± 28.77 (0–100)	**.000**†
General foot health	56.05 ± 24.92 (0–100)	57.66 ± 23.65 (0–100)	54.44 ± 26.08 (0–100)	.329†
General health	68.02 ± 22.95 (0–100)	79.50 ± 30.00 (10–100)	56.55 ± 19.96 (0–100)	**.000†**
Physical activity	84.52 ± 20.59 (5.56–100)	84.26 ± 20.47 (5.56–100)	84.79 ± 20.74 (5.56–100)	.221†
Social capacity	78.82 ± 24.33 (0–100)	82.52 ± 20.06 (0–100)	75.11 ± 27.50 (0–100)	.025†
Vitality	54.73 ± 23.25 (0–100)	53.32 ± 25.28 (0–100)	56.14 ± 20.99 (0–100)	.048†

^†^Median ± IR (range) and Mann–Whitney U test were utilised.

In all the analyses, *p* < .05 (with a 95% confidence interval) was considered statistically significant.

The differences between the groups were statistically significant for footwear and general health and there were no significant differences for the dimensions of the questionnaire that assessed foot pain, foot function, general foot health, physical activity, social capacity and vitality.

Table 3 shows the multivariate logistic regression, where it can be observed that those people who had worse scores on the scale items related to footwear, physical activity, foot health and vitality, had a higher probability of being a case. This association was greater for footwear (OR 4.470 (IC95% 2.569–7.775)) followed by physical activity. However, general health showed a negative association with the probability of being a case or a control.

**Table 3:** Factors that affect the presence of pathologies in the feet according to the items of the scale used.

Variables of the equation*	Odds ratio* (95% IC)	*p* value
**Foot pain (1)**		
0–81.25	1	.86
> 81.25	.619 (.359–1.070)	
**Foot function (1)**		
0–87.5	1	.387
> 87.5	1.284 (.729–2.263)	
**Footwear (1)**		
0–50.00	1	**.000**
> 50	4.470 (2.569–7.775)	
**General foot health (1)**		
0–60	1	.042
> 60	1.902 (1.024–3.535)	
**General Health (1)**		
0–70	1	**.000**
> 70	.040 (.022–.073)	
**Physical activity (1)**		
0–94	1	**.000**
> 94	3.281 (1.803- 5.970)	
**Social Capacity (1)**		
0–87.50	1	.735
> 87.50	1.100 (.632–1.917)	
**Vitality (1)**		
0–50	1	.020
> 50	1.850 (1.103—3.104)	

*Adjusted Odds Ratio by sex, age and BMI.

(1) Poor Health Status.

*The median value has been used for the cut off points.

## Discussion

The results of this research show that people with foot pathologies have a worse quality of life than the general population. This is observed with the assessment of the items on the scale used, both globally and individually. The analysis of the individual items showed that the people with the worst scores in the use of footwear were up to 4 times more likely to have foot pathologies compared to controls. To our knowledge, this is the first study to analyse the quality of life of Spanish patients using the Foot Health Status Questionnaire, in its Spanish version.

The reason for carrying out this study is the high prevalence of foot pathologies in Europe, as reflected by Burzykowski et al. in a multicentre project involving 70,497 patients who presented ranges of 56 to 64% of various pathologies in the feet with and without infection, where they confirm that early diagnosis prevents long-term structural or infectious sequelae, morbidity and cost associated with foot care^17^. In addition, several existing studies indicate lower satisfaction in quality of life related to foot health in people diagnosed with foot problems and the presence of systemic diseases^18–23^.

However, based on our knowledge, there is a lack of studies that analyse the quality of life related to foot health in the general population diagnosed with foot problems without the presence of associated risk factors and without the presence of other systemic diseases that may influence the negative impact on general health and specifically on the foot. In this way, the findings of our study are the first to reveal that foot pathologies in people who do not have systemic diseases negatively influence quality of life compared to a group of healthy people, presenting lower scores in the domain related to footwear.

These data are consistent with previous studies conducted at different stages of life that reflect the negative impact of quality of life and its relationship with the feet^8,24–26^.

For this reason, regular visits to the doctor and podiatrist are key aspects for improving foot health and health at a general level, being a predictor factor in optimising health spending and contributing to the improvement of systemic diseases, which individuals can present at different stages of life^8,27^.

In addition, the study shows how people with foot pathologies record a lower value in the footwear domain score, which is measured specifically in the first section of the questionnaire and is consistent with the prospective study carried out by Bennet et al. that evaluated the quality of life related to foot health in a 6-month prospective study conducted in a sample of 140 people with foot pathologies, with a mean age of 48.3 years, before and after undergoing surgery on the foot^28^. Gilheany et al. in a prospective study in 122 patients with a mean age of 48 years, who attended the pre-surgical consultation for presenting hallux valgus and hallux rigidus, showed low values ​​in the footwear domain, coinciding with the results of our investigation^29^.

Regarding the second section of the questionnaire, the dimensions of vitality and physical activity are lower in the case group, as is the case–control study carried out by López-López et al. in a sample of 150 patients with a mean age of 49.5 years with keratotic foot problems^30^. The case–control study carried out by Irving et al. in 94 patients, with a mean age of 52.3 years with chronic pain in the heel, showed similar results in the case group to those obtained in our study^6^.

There are several limitations to this study. The main one consists of having selected controls who attended the same podiatric clinic. It is likely that if controls from some other location had been included, the differences would have been greater, because although the controls did not have foot pathologies, they could have had them recently or they could have been worried about having them and therefore have decided to go to the clinic even though they were healthy. Another limitation resides in having carried out the research in a single clinic, since some external validity is subtracted from the results obtained. Future studies should include a larger number of participating centres. In addition, case and control groups were not matched-paired by sex, age nor BMI, the multivariate logistic regression was adjusted for sex, age, and BMI and were provided as ORs with 95% confidence intervals.

Among the advantages is the fact of having used a validated questionnaire to collect data on foot problems and quality of life, which makes it possible to use a reliable measuring instrument and also to be able to compare the results obtained with those of other investigations that have used the same questionnaire. Another additional advantage is the relatively high number of patients included, almost half a thousand, which allows obtaining relatively reliable estimates. The fact of having comparable subjects in terms of sex and age is also an advantage, since the observed differences will not be explained by imbalances in these variables.

Finally, the results presented in this research highlight the need to continue investigating the impact caused by alterations and deformities of the feet on quality of life, with the aim of optimising therapeutic interventions related to the feet prescribed by podiatrists and doctors in search of improvements in people’s health, well-being and autonomy.

## Conclusions

Foot problems have a negative impact on quality of life related to foot health, where the domains of footwear, general health and physical activity seem to be the factors that are associated with the presence of alterations and deformities in the feet.
